# Specificity of the female’s local cellular immune response in genital plug producing scorpion species

**DOI:** 10.1371/journal.pone.0208682

**Published:** 2019-02-11

**Authors:** Mariela A. Oviedo-Diego, Camilo I. Mattoni, Alfredo V. Peretti

**Affiliations:** 1 Universidad Nacional de Córdoba, Facultad de Ciencias Exactas, Físicas y Naturales, Departamento de Diversidad Biológica y Ecología, Córdoba Capital, Córdoba, Argentina; 2 Consejo Nacional de Investigaciones Científicas y Técnicas (CONICET), Instituto de Diversidad y Ecología Animal (IDEA), Laboratorio de Biología Reproductiva y Evolución, Córdoba Capital, Cordoba, Argentina; Uppsala Universitet, SWEDEN

## Abstract

Immune defense is a key feature in the life history of organisms, expensive to maintain, highly regulated by individuals and exposed to physiological and evolutionary trade-offs. In chelicerates, relatively scarce are the studies that relate postcopulatory mechanisms and immune response parameters. This work makes an approximation to the female’s immunological consequences produced after the placement of a foreign body in the genitalia of three scorpions species, two species that normally receive genital plugs during mating (*Urophonius brachycentrus* and *U*. *achalensis*) and one that does not (*Zabius fuscus*). Here we performed the first morphological description of the natural plugs of the two *Urophonius* species. We described complex three zoned structure anchored to the female genital atrium and based on this information we placed implants in the genitalia (for eliciting the local immune response) of virgin females of the three species and measured the immune encapsulation response to this foreign body. We found a greater and heterogeneous response in different zones of the implants in the plug producing species. To corroborate the specificity of this immune response, we compared the local genital reaction with the triggered response at a systemic level by inserting implants into the female body cavity of *U*. *brachycentrus* and *Zabius fuscus*. We found that the systemic response did not differ between species and that only in the plug producing species the local response in the genitalia was higher than the systemic one. We also compared the total hemocyte load before and after the genital implantation to see if this parameter was compromised by the immunological challenge. We confirmed that in *Urophonius* species the presence of a strange body in the genitalia caused a decrease in the hemocyte load. Besides, we find correlations between the body weight and the immunological parameters, as well as between different immunological parameters with each other. Complementarily, we characterized the hemocytes of the three scorpion species for the first time. This comparative study can help to provide a wider framework of the immunological characteristics of the species, their differences and their relationship with the particular postcopulatory mechanism such as the genital plugs.

## Introduction

Arthropods have a relatively simpler immune system than vertebrates since they lack acquired immunity ([1] but see [2]), although this does not mean that the immune system is less specific [3–5]. Immune responses comprise cellular-like responses mediated by the hemocytes -granulocytes (GRs) and plasmatocytes (PLs)- (e.g., coagulation, phagocytosis, nodule formation, encapsulation), and humoral-mediated responses (e.g., complement-like proteins, antimicrobial peptides, products generated by the phenoloxidase pathway) [6–8]. In particular, the encapsulation response (i.e., hemocytes’ adhesion in tight layers around an extrinsic factor) involves the action of GRs that recognize the extrinsic factor and release granules (with chemical signals of recruitment of PLs, enzymes, and precursors for melanin synthesis and ‘encapsulation-promoting factors’) [6, 9]. Therefore, the capsule formation is associated with melanization produced by the prophenoloxidase (proPO) cascade (activation of the phenoloxidase enzyme) with reactive oxygen and nitrogen species emitted and targeted against the extrinsic factor [10].

During infection, a systemic cellular immune response is mounted, and a large set of antimicrobial peptides (AMPs) are produced and secreted to the hemolymph [11]. In arthropods, a local immune response was also described where immune genes are expressed in the epidermal cells under the cuticle (epidermis, reproductive system, respiratory and digestive tract) that contact with the exterior and potential infectious microorganisms [11–15]. This type of response would be analogous to vertebrate mucosal immunity, and apparently, local and systemic immunological gene expression would be regulated by different signaling pathways [13].

Several studies have found changes in the immune system after mating [16]. For example, it has been found that, after mating, some immunological parameters may be improved as in the case of the crickets *Gryllus texensis* (Orthoptera, Gryllidae) where mated females have higher parasite resistance than virgins, or *Allonemobius socius* (Orthoptera, Trigonidiidae) wher eas mating frequency increases, hemocyte load, lytic activity, and encapsulation ability [17–18]. Conversely, in many cases, the immunological parameters can be weakened [18–22] as found in the beetle *Tenebrio molitor* with a reduction in phenoloxidase activity after mating [20]. Also, mating may cause the activation of immune system molecules in reproductive tissues [12, 23] or changes in the expression of immunity genes [24]. In the framework of the theory of immunocompetence, higher quality individuals are better able to meet the costs of maintaining good sexual characters and good immunological defense, and will, therefore, be preferred as couples [25–26].

Among the many reproductive strategies that organisms exhibit, there are some that involve males’ adaptations favored by sperm competition to increase their reproductive success [27–29]. Males would compete for the monopolization of females toward preventing, reducing or avoiding sperm competition [28, 30]. Genital plugs are structures that block or cover some portion of the female genitalia after mating and consequently can prevent sperm competition [30]. The plugging of females is a widespread phenomenon in the animal kingdom, including insects and arachnids [30–34]. Within arachnids, genital plugs are morphologically diverse, varying according to the taxa. In general, genital plugs can be formed by the coagulation of the male’s ejaculate or glandular substances [35–41], portions of the spermatophores [31, 42–43] or even parts of the male’s body or genitalia [44–46]. However, because the plugs could represent the result of sexual conflict in the domain of fertilization [47–48], sexually antagonistic coevolution would favor counter-adaptations of the females. For example, females can prevent the placement of a plug [49–51], controlling the duration of the mating [52–53] or actively removing it or degrading it [54–60]. Female control of the fate of the genital plug has been proposed as a mechanism of cryptic female choice [60–61]. The post-mating physiological consequences that are triggered in the female after the deposition of the male’s genital plug have not been well studied. Some studies have described secretions of the epithelium of female genitalia that adhere material to the plug and may help to anchor or consolidate the genital plug, or conversely, this material may degrade the male’s plug [35, 62–63].

In chelicerates, some studies investigate hemocyte ultrastructure [64–65] and the presence of antimicrobial molecules in the hemolymph [66–68]. However, we still need studies that evaluate the differences between the systemic and local immune responses and the relationship between postcopulatory mechanisms and immune response parameters. The reproductive biology of scorpions has certain characteristics that make it a potentially useful model for the study of these topics. Their courtship is complex and ritualized, after which males adhere a sclerotized spermatophore to the soil, from which the female receives the sperm [69–71]. The sperm penetrates the genital aperture of female and advances through the genital atrium towards the seminal receptacle [72]. After fertilization, the viviparous embryos develop within the ovariuterus until the time of parturition [73–74]. In many species of scorpions, females present a genital plug after the sperm transfer [32], although their morphology is very diverse and the function is discussed [31]. In some cases, the formation of the genital plug is almost completely attributed to the male [32, 39, 43], although the participation of the female has been suggested [31–32, 39, 75–76]. In ultrastructural studies of the atrial epithelium of the female, pores and glandular cells and high secretory activity have been described, so the possibility of female participation is strongly expected [38, 76–77].

In this work, females of three scorpion species are compared. *Urophonius brachycentrus* (Thorell, 1877) and *U*. *achalensis* (Ábalos and Hominal, 1974) [31, 78] two species of the family Bothriuridae that have genital plugs were compared with *Zabius fuscus* (Thorell 1877), a buthid species with no genital plug [32]. *Urophonius* has a ‘mixed’ plug with a combination of detachable portions of the spermatophore and glandular substances [31]. It is known that males of *Urophonius brachycentrus* and *U*. *achalensis* transfer an ‘initial plug’ formed by two hemi-mating plugs (one per hemispermatophore) that join in the formation of the spermatophore during sperm transfer. This ‘initial plug’ presents a translucent coloration when it has just been transferred to the female and shows a progressive darkening during the reproductive season (see below the results of this study). Certain changes in the size and coloration of the plugs of *Urophonius*, and other species [31, 39, 43], might be linked to cellular immunological responses such as encapsulation and melanization. These reactions strongly resemble the systemic cellular immune response that is activated on artificial implants (e.g., nylon filaments) that are inserted into the hemocoel of individuals and that, after a time, present areas with dark encapsulations [79–82].

To elucidate the immunological mechanisms triggers by the female against a foreign body in the reproductive tract, we first described the genital plugs and their positioning within the female genital atrium in both *Urophonius* species to begin by approaching a foreign body which the females face naturally. In second place, we evaluated and compared the systemic and local cellular immune response of the species placing implants in the body cavity and in the reproductive tract respectively. We expect not to observe interspecific differences in the systemic cellular immune reaction, but in the local response to a foreign body with a higher local immune response (larger, dark-colored encapsulations) in the plug producing species compared with the non-plug species. In turn, we expect to observe differences between the systemic and local immune responses in *Urophonius*, and not to observe these differences in *Z*. *fuscus* that does not have a genital plug. Thirdly, we surveyed the types of hemocytes present in the hemolymph and total hemocyte load (THL) of the females of each species. We observed if there were changes in the THL before and after the placement of the genital implants, in search of possible connections between the triggering of the local and systemic immune response. Finally, we looked for relations between the immunological parameters (encapsulation areas / coloration as a proxy of melanization and THL) and between the parameters and body weight of individuals, since heavier females could have greater THL and be more competent to face an immune challenge such as the genital implant.

## Materials and methods

### Studied species, collection and rearing

Two sister species of the family Bothriuridae were studied. *Urophonius brachycentrus* and *U*. *achalensis* present winter surface activity [83] and were collected at the beginning of the season (from May to June). The time of collection was determined to ensure that the females were virgins and did not have a genital plug, as when the females are inseminated they always present a genital plug, and their distal portion is visible below the genital operculum (S1A Fig). In contrast, *Zabius fuscus* individuals are active in summer (November to March) [84], and inseminated females do not present genital plug [12, 18]. Individuals were collected during the day by turning rocks over in the Sierras Grandes at altitudes from 800 to 2000 MASL (*U*. *brachycentrus*: 31°23'30.7"S 64°43'42.1"W; *U achalensis*: 31°21'52.9"S 64°46'33.6"W; *Z*. *fuscus*: 31°22'42.5"S 64°35'28.7"W). Although *Zabius fuscus* (Fam. Buthidae) is phylogenetically distant from the two species of Bothriuridae, it was chosen for this study because as far as we know, there are no bothriurid species that do not have a genital plug [31–32, 38, 85–87]. In the laboratory, each specimen was weighed with a digital balance (Ohaus Pioneer PA114). The scorpions were conditioned in individual plastic containers (9 cm x 6 cm) and were kept with moistened cotton as a water supply, and fed once a week with larvae of *Tenebrio molitor* (Coleoptera, Tenebrionidae) or adults of *Shelfordella tartara* (Blattodea, Blattidae). We maintained the specimens at constant temperatures (10°C in winter, 25°C in summer). Voucher specimens were deposited in the collection of Laboratorio de Biología Reproductiva y Evolución, Universidad Nacional de Córdoba, Argentina.

### Morphology of genital plugs and positioning within female

We performed dissections to observe the positioning of the plug within inseminated females (*U*. *brachycentrus* n = 20; *U*. *achalensis* n = 20). The specimens were sacrificed in a freezer at -20°C for fifteen minutes and then dissected under a stereoscopic microscope (Nikon SMZ 1500). After the dissection, we removed the genital plug from the female’s atrium with straight tweezers. The dissected specimens and genital plugs were photographed with a digital camera (Nikon Digital Sight DS-FI1-U2) coupled to the stereoscopic microscope. Some genital plugs (n = 10 per species) were kept in a 1 mL microcentrifuge tube exposed to the air and were photographed every week for a month. In this way, we observed if there were changes in the coloration or morphology of the genital plugs outside the female (e.g., by oxidation of the plug material). We also evaluated changes in the coloration and morphology of the plugs inside the females throughout the reproductive season (n = 10 per species) by examining the external portion of the plugs below the genital operculum.

### Systemic and local cellular immune response to a foreign body

#### Placement and removal of implants

The foreign body consisted of a piece of sterile monofilament (3 mm x 0.1 mm) (nylon monofilament for surgical suture -polyamide 6-) (from now on ‘implant’) (S1E Fig). To elicit the systemic cellular immune response of encapsulation, we placed implants inside the mesosoma piercing the dorsal pleural membrane between the fifth and sixth segment (in *Z*. *fuscus* n = 8 and *U*. *brachcyentrus* n = 11) (S1C Fig). The specimens were immobilized on a microscope slide with Parafilm. A small hole was made in the parafilm to access the zone of implantation, we piercing the dorsal pleural membrane with a fine headless sterilized entomological pin and we inserted gently the implant in the body cavity. In the same way, to elicit the local cellular immune response we placed implants in the genital atrium of each female, resembling in size and positioning the genital plug present in females of the *Urophonius* species (in *U*. *brachcyentrus* n = 10, *U*. *achalensis* n = 10 and *Z*. *fuscus* n = 10) (S1B and S1G Fig). For this, we inserted the implant up to the end of the genital atrium by lifting the female genital operculum with straight tweezers. The surface of the implants was slightly roughened with sandpaper to reach a rough surface and enhance the adhesion of hemocytes to the implants [88–89]. The implantation procedure does not cause any damage to the female. The implants were left inside the female for 30 days since it has been observed that the genital plug of *Urophonius* takes approximately this time to present some darkening (Oviedo-Diego, Mattoni, Peretti personal observations). After this period, the implants were carefully removed, and the tissue remnants were cleaned. Each implant was photographed from two perspectives (front and back), rotating 180° [90] with a digital camera (Nikon Digital Sight DS-FI1-U2) coupled to a stereomicroscope (Nikon SMZ1500). We used a photographic protocol that kept light exposure and magnification constant. Then the implants were preserved in ethanol 80%.

#### Area and coloration of the encapsulations on implants

The areas of encapsulations on each implant were measured by processing the images with ImageJ 1.45 software [91]. For statistical analysis, we divided the implants into three zones. It is known that the genital plugs of the *Urophonius* species studied also have three zones (‘distal’, ‘middle’ and ‘proximal’ to the body of the individual) (See Results) (S1D Fig). The zones of the implants were defined by dividing the total length of the filament (3 mm) into three parts of equal length so that each zone was 1 mm long (S1E Fig). The zone that remained within the body of the female was the ‘proximal’ zone of the implant, while the zone more distal to the female’s body was the ‘distal’ zone of the implant. We compared the areas of the encapsulations between the zones of the implants, between species and between the type of immune response elicited (implants placed in the genitalia–‘genital implants’-: eliciting local immune response; implants placed in the body cavity: eliciting systemic immune response). We classified the encapsulations as melanotic (ME) or non-melanotic (NME) according to their coloration. This coloration was calculated with the average grayscale value from the pixels of the different zones of the implant encapsulations. The 0 value represents black and 255, white. The classification of encapsulations coloration was carried out using a threshold value of 50 in the average grayscale, being ME if the color was lower than the threshold value and NME if it was higher than this value.

### Extraction, characterization and quantification of hemocytes

We completely excised the second left leg of individuals (between tarsus and tibia) to allow a considerable drop of hemolymph to flow from the wound. Immediately, we took a sample of 0.75 μl of hemolymph with a glass capillary from the wound [92]. This sample was mixed with 9.25 μl of Spider Saline Solution [93] in a microcentrifuge tube. We performed a five-second pulse of vortex three times to the solution to homogenize the sample. Immediately after, we placed the sample in a Neubauer chamber for counting under a light microscope with a phase contrast objective 100x (Nikon Eclipse 50i) [94] at 400X. All hemocytes from virgin females of the different species were identified and counted [95]. We performed the characterization of hemocytes by observing and photographing their morphological characteristics with a digital camera coupled to the microscope (Nikon Digital Sight DS-FI1-U2). The total hemocyte load (THL) (number of hemocytes per milliliter of hemolymph) was compared in two stages: before the genital implantation and after extraction of the genital implant (through a second cut of the same leg).

### Statistical analyses

We analyze the data with generalized linear mixed models (GLMM). In the analysis of the implants encapsulations, the variables response were the ME and NME areas (mm^**2**^) and the average grayscale value of each type of encapsulation (coloration). The zone of the implant (‘distal’, ‘middle’, ‘proximal’), the type of immune response elicited (systemic vs local), the species (*U*. *brachycentrus*, *U*. *achalensis*, *Z fuscus*), and the individual’ body weight were the fixed effects. The body weight of the individuals was measured before and after the placement of the implants and since there were no significant differences between both instances (Mann–Whitney U test; Z = 0.409, p = 0.683) the average weight value for statistical analyses was considered. In the quantification of hemocytes, the variable response was the THL, and the fixed factors were the species, the stage of quantification (before and after the placement of the genital implant) and the body weight of the individuals. We also evaluated the possible interactions between the fixed factors analyzed. We included the individuals’ identity in all the models as a random effect. If the random effect variance was small, the effect of the random variable was discarded. Normality and homogeneity of variances of the variables were assessed graphically and analytically. If the assumptions were not met, the variable according to the best distribution was modeled. The coloration of ME and the THL presented a normal distribution and were analyzed with the package lme4 [96]. The areas of ME and NME and the coloration of NME presented a gamma distribution, so they were modeled using the glmmadmb function [97]. We used and lsmeans [98] for a posteriori tests in R v. 3.3.3 64 bit [99]. Also, multiple correlations with the Spearman’s method were performed between the different immunological parameters including the three species of scorpions together, and between these parameters and the individuals’ body weight. A significance level α of 0.05 was considered.

## Results

### Morphology of genital plugs and positioning within female

**Fig 1:**
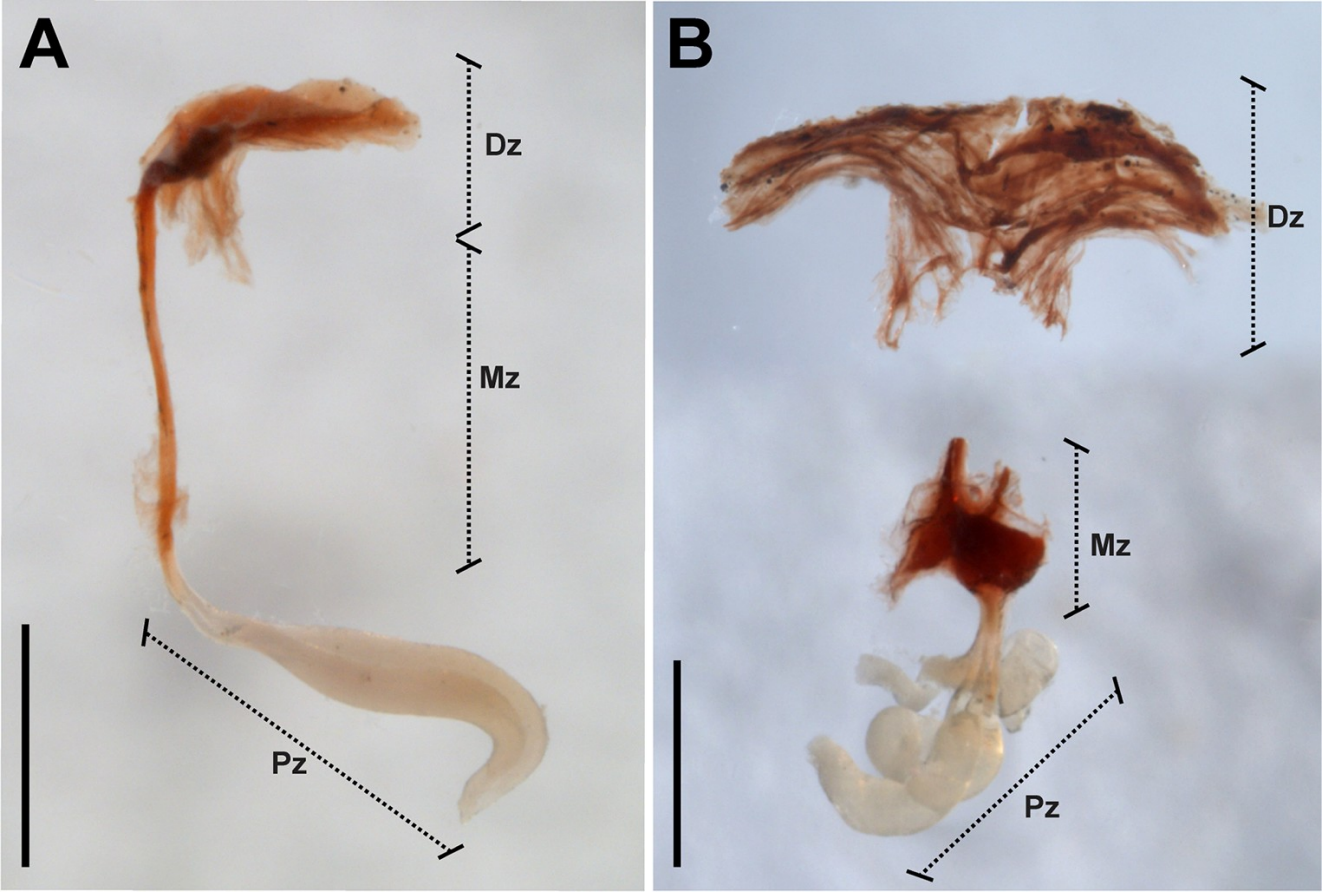
Genital plugs of *Urophonius* species. (A) Genital plug of *Urophonius brachycentrus*. (B) Genital plug of *Urophonius achalensis*, note that the plug is shown severed under the distal zone due to handling during extraction. Abbreviations: Dz, distal zone; Mz, middle zone; Pz, proximal zone. Scale bars: 1 mm.

In both species, the genital plug adjusted exactly to the female’s atrium and blocked the lumen and the genital aperture (S1A and S1F Fig). The genital plugs presented three double-shaped zones (Fig 1 and S1D Fig). The ‘distal zone’ to the individual’s body was visible from the outside and extended below the genital operculum covering the genital aperture (S1A Fig). It was always sclerotized, brittle and darkly colored. In *U*. *brachycentrus* this zone resembled two thin ‘wings’ (Fig 1A). In contrast, in *U*. *achalensis*, this zone was wider with concave platform shape towards the genital aperture (Fig 1B). Next to the ‘distal’ zone was the ‘middle’ zone, also sclerotized and dark, formed by two fused structures running along the lumen of the female atrium. While in *U*. *brachycentrus* this zone was thin and long, in *U*. *achalensis* it was shorter, and it was sometimes more difficult to distinguish the fused structures (Fig 1 and S1D Fig). Finally, the ‘proximal’ zone consisted of one or more sacciform globular structures, with a flexible gelatinous consistency and a white-yellowish coloration. Projections ascended from the end of the genital atrium to the duct of one of the spermathecae, sometimes occluding the duct (S1F Fig). In *U*. *brachycentrus* two projections were always found in the ‘proximal’ zone, while in *U*. *achalensis* the number was variable from one to four proximal projections. We found that the plug undergoes changes in coloration and morphology over time in the genitalia of the female. After mating the plugs presented the ‘distal’ zone (visible below the operculum) with translucent coloration and thin, fragile consistency. As the reproductive season progressed, the plug darkened and acquired a sclerotized consistency (June to August). We observed a decrease in the size of the distal zone of the plug towards the end of the season before parturition (November to December). Conversely, no changes were observed in the coloration or morphology of the plugs extracted from the females and exposed to the air.

### Types of encapsulations observed on implants

**Fig 2:**
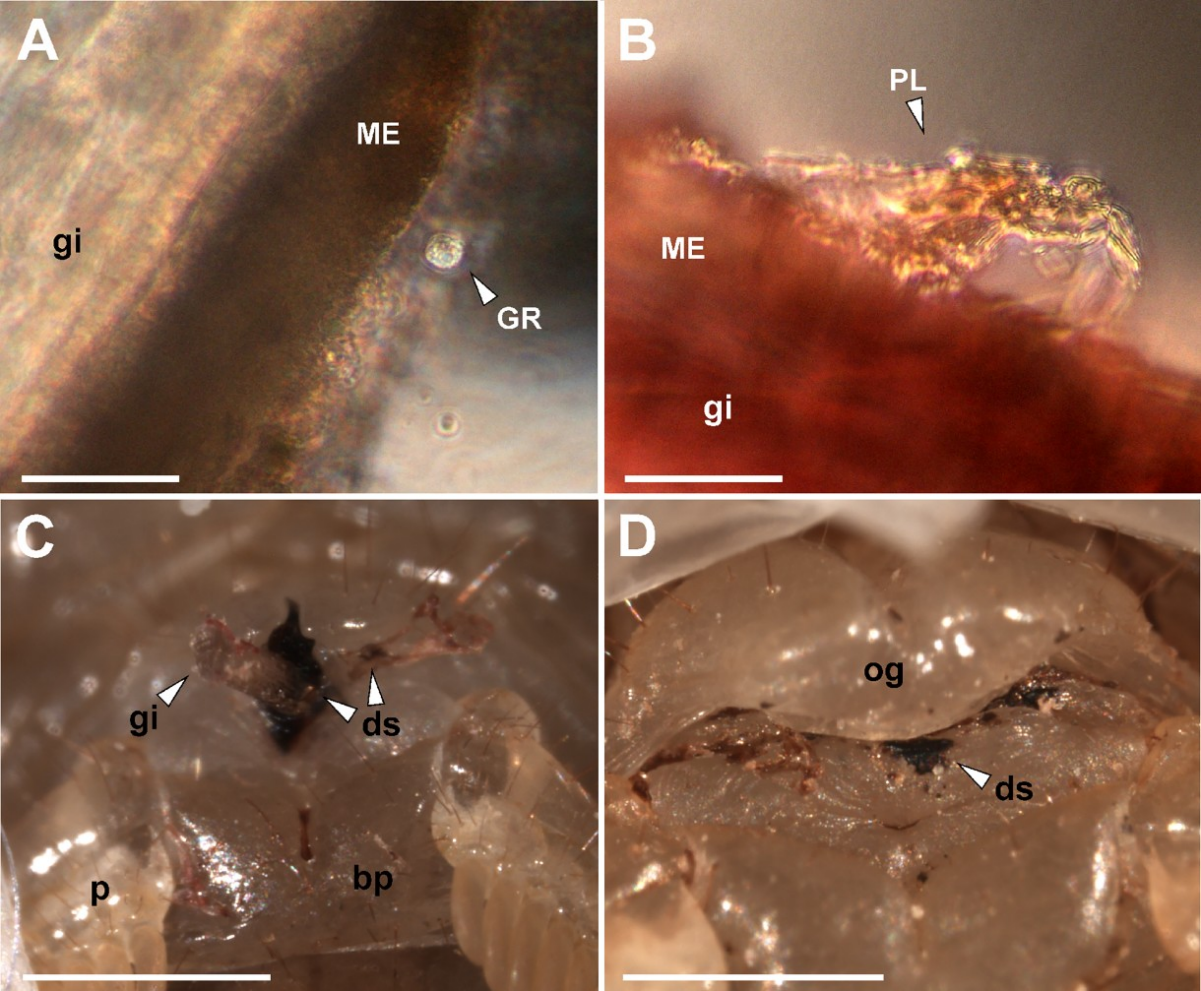
Hemocytes cells and distal substances found on the implants of females of *Urophonius*. (A)(B) Surface of the genital implant of *Urophonius brachycentrus* under an optical microscope, note hemocyte cells. (C)(D) External female genitalia of *Urophonius achalensis*, note the genital implant and some surrounding substance in the area of the genital aperture. Abbreviations: bp, basal piece; ds, distal substance produced by the female; gi, genital implant; GR, granulocyte; ME, melanotic encapsulation; og, genital operculum; p, pectine; PL, plasmatocyte. Scale bars: A = 0.2 mm; B = 40 μm; C, D = 1 mm.

Different characteristics and magnitudes of encapsulation response were observed, depending on the species, the type of immune response elicited, and the zone of the implant (S1 Table and S2 Fig). Occasionally, a non-melanotic encapsulation (NME) response was observed, generally with excrescences almost continuously surrounding the genital plug. This type of encapsulation was white-yellowish, translucent or opaque. Melanotic encapsulation (ME) presented more specific arrangements, generally in the form of isolated granules in different zones. All the implants were encapsulated, although in some cases the encapsulations were not present in all the zones of the implant. Hemocytes could be observed on the implants and in their surroundings under an optical microscope (Fig 2A and 2B). In the case of genital implants, sometimes it was possible to see the deposition of a substance around the entrance of the genital implant by the genital aperture, and sometimes we observed the formation of projections (Fig 2C and 2D).

### Local vs systemic cellular immune responses in non-plug producing species

**Fig 3:**
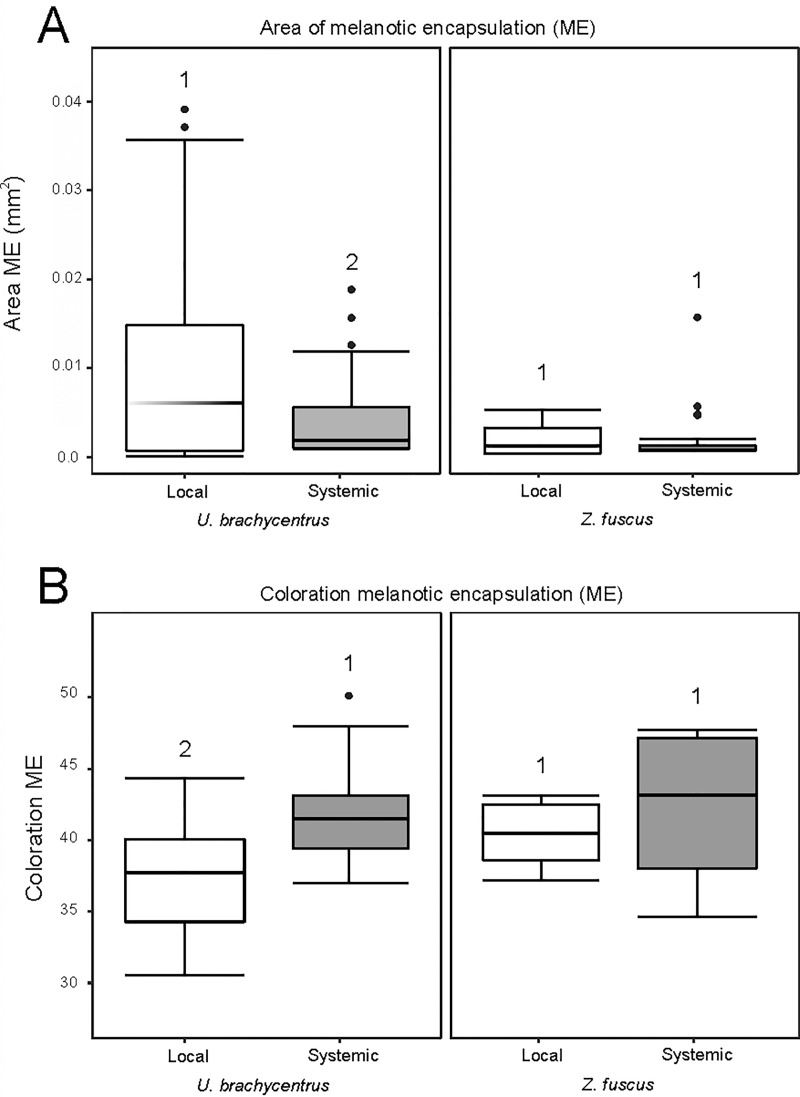
Graphs of local vs systemic immunological parameters in females of two scorpion species. Boxplots showing the distribution of data set and differences between the species and the type of response elicited (systemic: implants inserted in the body cavity; local: implants inserted in the genitalia) in females of *Urophonius brachycentrus* and *Zabius fuscus*. (A) Area of melanotic encapsulation (ME) (mm^2^). (B) Coloration of ME encapsulation (average grayscale value). Numbers above the boxplots indicate significant differences (p <0.05) between types of immune response elicited.

We observe cellular response in implants of both sites of implantation (genital atrium and body cavity), showing: NME encapsulations with transparent excrescences in all zones of the implant, and ME encapsulations located more restricted to certain portions of the implants (especially in the entrance of the implant into the body of the individual—distal zone of the implant) (S2A and S2C Fig). In *Z*. *fuscus*, we did not found a statistical effect of the type of immune response elicited in the areas of encapsulation (ME: Df = 1, χ^**2**^ = 0.197, p = 0.658; NME: Df = 2, χ^**2**^ = 0.222, p = 0.637) (Fig 3A). We found an effect of the zone of the implant: distal zones with greater ME values than other zones of the implant (Df = 2, χ^**2**^ = 75.973, p<0.0005), although there were no differences between the zones of the implant for the NME (Df = 2, χ^**2**^ = 0.552, p = 0.759). We also did not observe differences in the coloration of ME and NME encapsulations between the types of immune response elicited (ME: Df = 1, χ^**2**^ = 0.069, p = 0.791; NME: Df = 1, χ^**2**^ = 0.062, p = 0.803) (Fig 3B), nor between the zones of the implants (ME: Df = 2, χ^**2**^ = 4.512, p = 0.105; NME: Df = 2, χ^**2**^ = 2.216, p = 0.33). No relationship was found between the immunological parameters analyzed and the individuals' body weight (Area ME: p = 0.452, R^**2**^ = 0.115; Area NME: p = 0.359, R^**2**^ = -0.14; Coloration ME: p = 0.459, R^**2**^ = 0.199; Coloration NME: p = 0.496; R^**2**^ = 0.104).

### Local vs systemic cellular immune responses in plug producing species (*U*. *brachycentrus*)

A significant statistical interaction was found between the type of immune response elicited and the zone of the implant for the encapsulation areas (ME: Df = 2, χ^**2**^ = 32.655, p<0.0005; NME: Df = 2, χ^**2**^ = 11.672, p = 0.003) (Fig 3A). The implants eliciting a systemic immune response (placed in the body cavity) presented smaller areas of ME and NME in their distal zones, and smaller NME areas in their proximal zones compared to the implants eliciting a local immune response (placed in the genitalia) (S2B and S2D Fig). The implants of the body cavity presented a homogeneous response of ME and NME encapsulation, without differences in these parameters between zones of the implant. In turn, in genital implants, there were differences in the area of encapsulation between the zones of the implant. An effect of the type of immune response elicited was found in the ME coloration (Df = 2, χ^**2**^ = 6.205, p = 0.013) (Fig 3B), because the implants eliciting systemic immune response presented clearer coloration. We also observed an effect of the zone of the implant in the ME coloration, with the middle zone always being darker than the proximal and distal zones in all the implants (Df = 1, χ^**2**^ = 19.611, p<0.0005). There were no significant differences for the NME coloration between zones of the implant (Df = 2, χ^**2**^ = 0.326, p = 0.849) and between the type of immune response elicited (Df = 1, χ^**2**^ = 2.299, p = 0.129). We did not fond a relation of the analyzed immunological parameters and the body weight of the individuals (Area ME: p = 0.563, R^**2**^ = -0.076; Area NME: p = 0.081, R^**2**^ = -0.227; Coloration ME: p = 0.656, R^**2**^ = 0.067; Coloration NME: p = 0.652; R^**2**^ = -0.059).

### Interspecific comparison of the cellular immune response

#### Comparing systemic cellular immune response

The encapsulation of implants placed in the body cavity were not different between plug producing species (*U*. *brachycentrus*) and not-plug producing species (*Z*. *fuscus*) in area of encapsulations (ME: Df = 1, χ^**2**^ = 0.0002, p = 0.988; NME: Df = 1, χ^**2**^ = 0.799, p = 0.371) and ME coloration (ME: Df = 1, χ^**2**^ = 0.371, p = 0.542) (Fig 3). We found an effect of the species in the NME coloration (Df = 1, χ^**2**^ = 11.119, p = 0.001). The NME encapsulations were clearer in all zones of the implant in *Z*. *fuscus*. We did not observe significant differences between the zones of the implants in any of the immunological parameters of both species (Area ME: Df = 2, χ^**2**^ = 2.333, p = 0.312; Area NME: Df = 2, χ^**2**^ = 2.013, p = 0.366; Coloration ME: Df = 2, χ^**2**^ = 0.226, p = 0.635; Coloration NME: Df = 2, χ^**2**^ = 1.395, p = 0.498).

#### Comparing local cellular immune response

**Fig 4:**
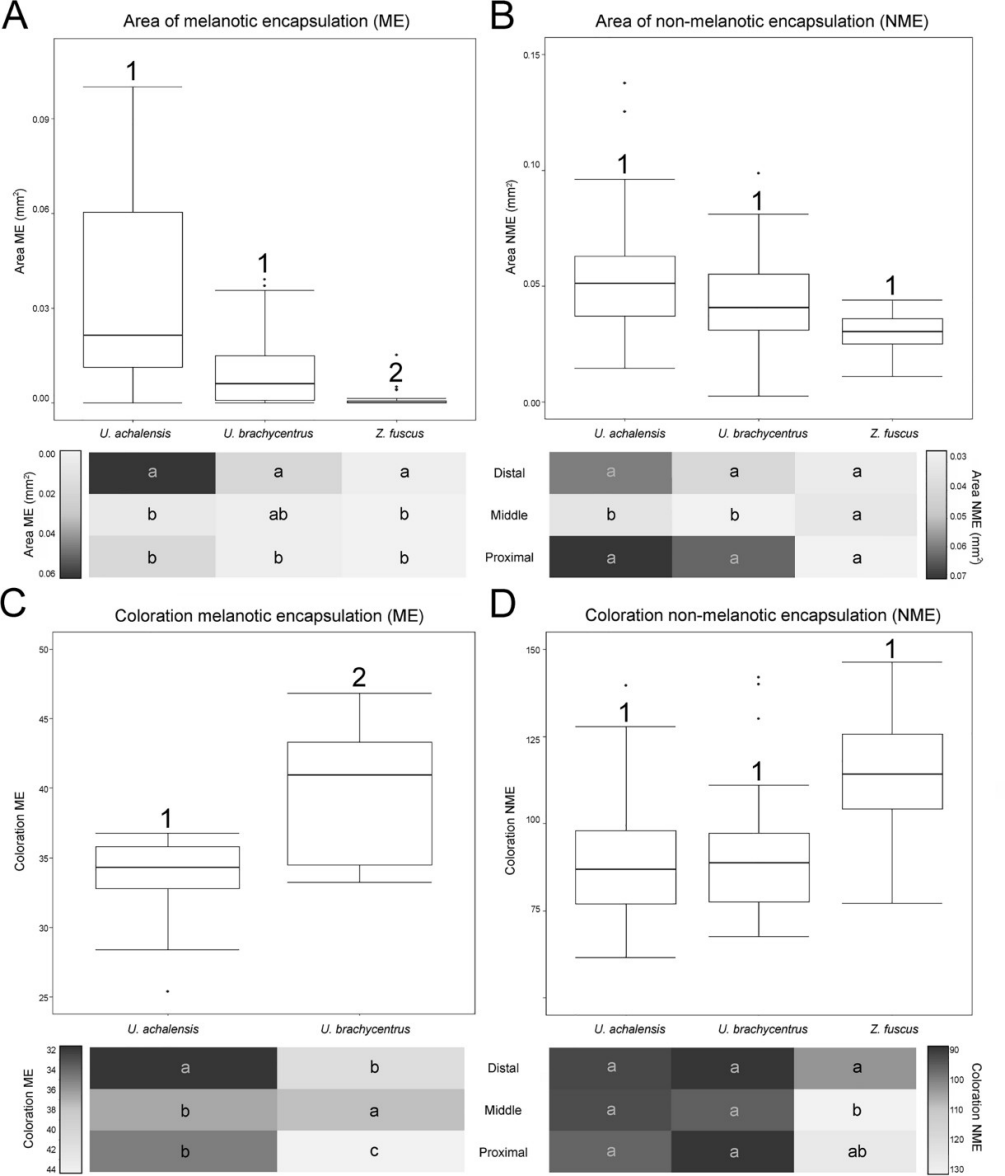
Graphs of local immunological parameters in females of three scorpion species. Top boxplots showing distribution of data set and differences between species, below heat maps charts in which average values are represented by colors (scale of reference of each variable to the side of the graph) according to the zones of the genital implant. (A) Area of melanotic encapsulation (ME) (mm^2^). (B) Area of non-melanotic encapsulation (NME) (mm^2^). (C) Coloration of NME encapsulation (average grayscale value). (D) Coloration of ME encapsulation (average grayscale value). Numbers indicate significant differences (p<0.05) between species above the boxplots. Letters on heat maps charts indicate significant differences (p <0.05) between zones of the genital implant within each species (i.e., intraspecific comparison: reading vertically, not horizontally).

The encapsulation response in the implants placed in the genitalia of the females was different between plug producing species (*U*. *brachycentrus*, *U*. *achalensis*) and not-plug producing species (*Z*. *fuscus*) and between the zones of the implants (Fig 4 and S2C and S2D Fig). In the area of encapsulation, we found an effect of the interaction between the species and the encapsulated zone of the genital implant (ME: Df = 2, χ^**2**^ = 14.827, p = 0.005; NME: Df = 2, χ^**2**^ = 20.411, p<0.0005) (Fig 4A and 4B). ME values for both species of *Urophonius* were higher than *Z*. *fuscus*. Differences were also found between the encapsulated areas of different zones of the genital implant. All species had higher ME in the ‘distal’ zone, although *U*. *brachycentrus* had fewer differences between zones. As for NME, all species presented similar areas of encapsulations. *Z*. *fuscus* showed a homogenous, low response in all zones, and *Urophonius* species showed a greater response in the ‘distal’ and ‘proximal’ zone. Since *Z*. *fuscus* females rarely presented ME in the ‘middle’ and ‘proximal’ zone of the genital plugs, only the coloration of these encapsulations was analyzed for the females of two *Urophonius* species. We found a significant interaction for ME coloration between the species and the encapsulated zone (Df = 2, χ^**2**^ = 29.698, p = p<0.0005) (Fig 4C). *Urophonius achalensis* females showed darkest encapsulations than *U*. *brachycentrus*. Also, *U*. *achalensis* presented ‘distal’ zones significantly darker than the rest of the zones. In contrast, *U*. *brachycentrus* presented darker ‘middle’ zones, followed by the ‘distal’ zones and significantly clearer ‘proximal’ zones. For the NME coloration, we found an effect of the interaction between the species and the implant zone (Df = 2, χ^**2**^ = 23.463, p<0.0005) (Fig 4D). *Zabius fuscus* presented in general clearer encapsulations although they were not significantly different from those of *Urophonius* spp. In *Z*. *fuscus*, the ‘distal’ zone was darker than the others. For the *Urophonius* species, all the zones presented NME encapsulations of similar coloration.

### Characterization of hemocytes of females of the studied species

**Fig 5:**
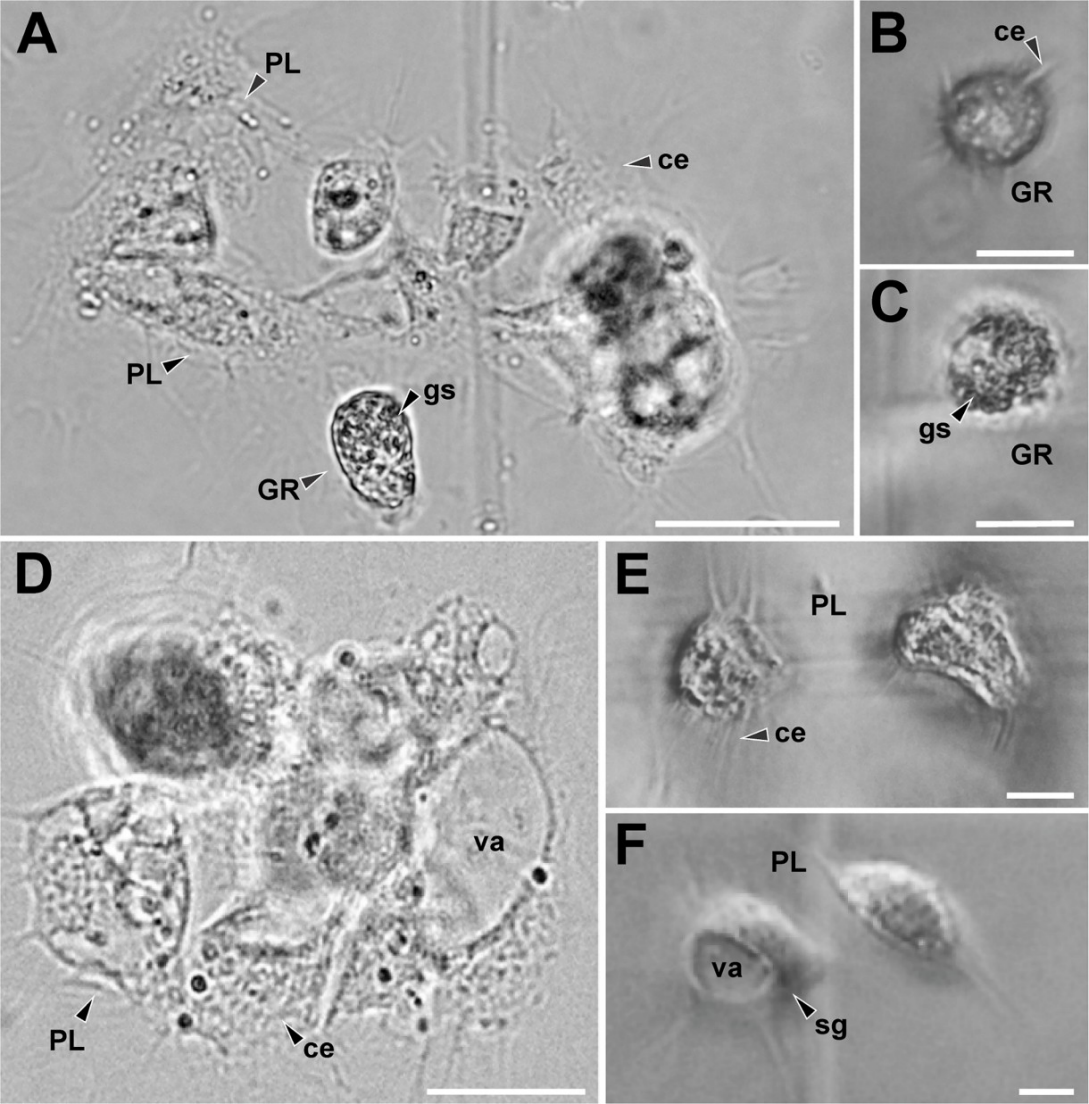
Hemocytes present in the hemolymph of females of three scorpions’ species. (A) Plasmatocytes (PLs) and granulocytes (GRs) cluster of *Urophonius brachycentrus*. (B) GRs of *Urophonius brachycentrus*, note isodiametric morphology, granules in the cytoplasm and short and acute cytoplasmic extensions. (C) GRs of *Zabius fuscus*, note granules in the cytoplasm and short cytoplasmic extensions. (D) PL cluster of *Urophonius achalensis*. (E) PLs of *Zabius fuscus* expanding their cytoplasmic extensions with small inclusions in their cytoplasm. (F) Signet-ring PL of *Urophonius brachycentrus*, note vacuole occupied a large portion of the cytoplasm of the cell. Abbreviations: ce, cytoplasmatic extensions; GR, granulocyte; gs, granules in the cytoplasm; PL, Plasmatocyte; sg, signet-ring plasmatocyte; va, vacuole. Scale bars: A, D = 50 μm; B, C = 20μm; E, F = 10 μm.

We identified different types of hemocyte cells in the hemolymph of the females in the species studied (Fig 5). Hemocytes presented different morphology and size. Granulocytes (GRs) were cells with spherical and isodiametric shapes and regular contours (Fig 5A–5C). GRs had cytoplasmic extensions variables in shape, although the extensions were in general short and acute. The cytoplasm of the GRs was dense and always presented abundant refractive oval shaped granules. Plasmatocytes (PLs) were highly variable in shape, generally spindle-shaped with multiple large, rounded cytoplasmic extensions radiating in ameboid form from the central zone (Fig 5A and 5D–5F). The cytoplasm was hyaline and homogenous, with vacuoles and small or no inclusions. Sometimes the vacuoles occupied a large portion of the cytoplasm of the cell, pushing the nucleus to an eccentric position, giving it a signet-ring appearance with sharp projections (Fig 5F). Also, other types of hemocytes were observed, although they were not found in all the samples. All these hemocytes had a rounded and rather an isodiametric shape and did not expand cytoplasmic extensions such as PLs. They presented granules in the cytoplasm of different shapes and nature. Cystocytes generally presented a cytoplasm with small granules, a large vacuole, and eccentric nucleus. Spherulocytes possessed large, dark granules or spherules of a homogeneous size, which completely obscured the nucleus of the cell. Adipohemocytes presented an eccentric nucleus with typical fat lipid droplets in their cytoplasm. Free cells were observed in the hemolymph and also grouped in agglomerates and, although in these cases it was difficult to identify the clustered cells, we were able to determine that on occasions the clusters may have cells of a different type (Fig 5A and 5D).

### Quantification of hemocytes before and after genital implantation

**Fig 6:**
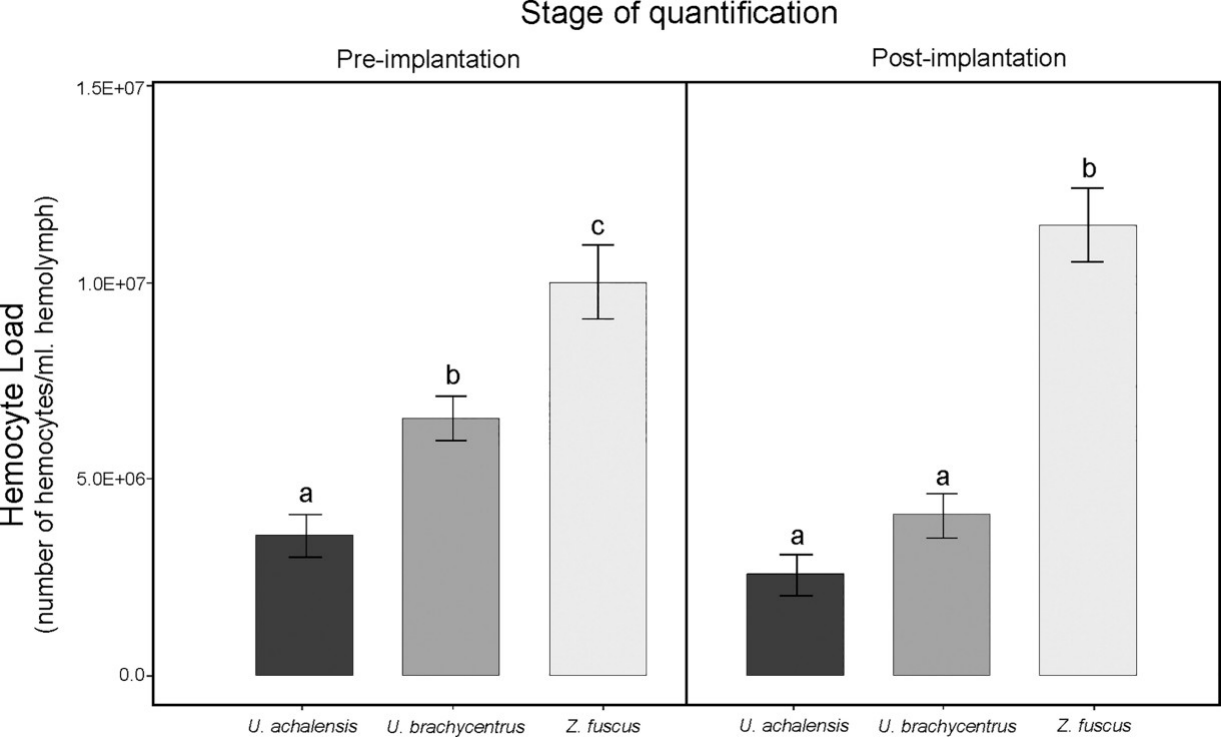
Total Hemocyte Load of females of the three scorpion species. Differences in total hemocyte load (THL) (number of hemocytes per milliliter of hemolymph) according to the stage of quantification (pre-implantation and post-implantation in genitalia) of three scorpion species. Letters indicate significant differences (p<0.05) between species. Values are shown in scientific E-notation where 'E' represents the exponential to 10.

We found a significant interaction between the species and the stage of quantification (Df = 2, χ^**2**^ = 35.364, p<0.0005) (Fig 6 and S1 Table). *Zabius fuscus* females showed the highest THL values both before and after the placement of the genital implants with respect to *Urophonius* females. The *Urophonius* spp. showed similar values of THL before the genital implantation. *Zabius fuscus* females showed no difference in THL between the two stages of quantification, while in both species of *Urophonius* females presented a decrease in THL after one month of the placement of the implant, with a greater decrease of *U*. *brachycentrus* than that of *U*. *achalensis* (Df = 1, F = 7.261, p = 0.015). Body weight had a positive effect on THL (Df = 1, χ^**2**^ = 7.708, p = 0.006).

### Correlations between immunological parameters of the local immune response

**Fig 7:**
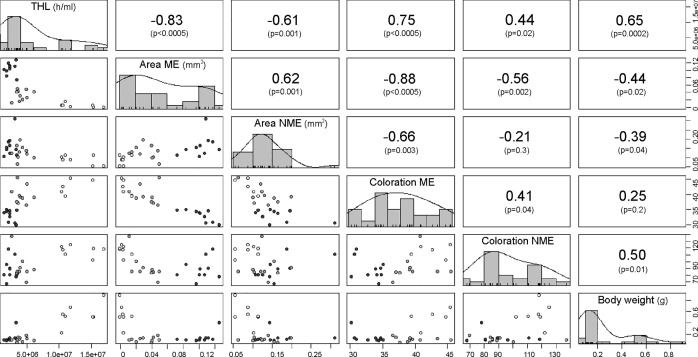
Correlation matrix between the individual’s local immunological parameters and body weight. In the diagonal portion of the matrix are found the parameters that were correlated with their corresponding frequency histogram: total hemocyte Load (THL) (number of hemocytes per milliliter of hemolymph h/ml); area of melanotic encapsulation (ME) (mm^2^); area of non-melanotic encapsulation (NME) (mm^2^); coloration of melanotic encapsulation (ME average grayscale value); coloration of non-melanotic encapsulation (NME average grayscale value); body weight (g). In the upper portion of the matrix are the Spearman correlation coefficients for each pair of variables and below the correlation p-value. In the lower portion of the matrix are graphs of dispersion of the variables, the colors of points represent the different species of scorpions (Black dots: *Urophonius achalensis*; Grey dots: *Urophonius brachycentrus*; White dots: *Zabius fuscus*). Abbreviations: ME, melanotic encapsulation of genital implant; NME, non-melanotic encapsulation of genital implant; THL, total hemocyte load. Some values are shown in scientific E-notation where 'E' represents the exponential to 10.

We noted that the immunological parameters of the local immune response, in general, were highly correlated with one another (Fig 7). A positive correlation was found between the female body weight and the THL, but a negative relationship was found between the body weight and the area of both types of encapsulations. Females with a higher THL also presented smaller ME and NME areas on the genital implants. However, the THL was correlated with the coloration of the encapsulated (i.e., clearer encapsulations). We noted a negative correlation between the area of ME and the coloration of the encapsulated zones, as well as between the NME area and the coloration of the ME encapsulations. We observed a positive correlation between the areas of both types of encapsulation.

## Discussion

Here we report the cellular immune response at a local and systemic level in females of three scorpion species. The study of the local immune response of encapsulation in the genitalia acquires relevance because females of two evaluated species face the placement of a natural genital plug (‘initial plug’) by the male. We have described for the first time the genital plug morphology of the *U*. *brachycentrus* and *U*. *achalensis*, and that allowed us to perceive a complex three zoned structure anchored to the female genital atrium. We also observed changes in the coloration of plugs over time, probably attributable to a female’s role. Given the need for experimental approaches to answer questions about the function or origin of genital plugs [31–33], we placed genital implants in this study to evaluate the female’s local immune cellular reaction to a foreign body in their genitalia. We found that females deposited secretions on the implants and that the zones had encapsulations of different coloration. We confirmed that females of plug producing species have a higher local immune response than the non-plug producing species. The comparison between the systemic and local cellular immune response between two scorpion species allowed corroborating the specificity of the local immune response of the plug producing species. We confirmed that in *Urophonius* species the presence of a strange body in the genitalia caused a decrease in the hemocyte load. These results could suggest a possible female role in the plugs’ formation in *Urophonius* and may help to provide a wider framework of the different physiological consequences related to this post-copulatory mechanism.

### Surveying the specificity of the local cellular immune response in plug producing species

The immune system of the female genital tract epithelium has evolved by making contact with sperm, with male's ejaculate substances and with potentially infectious agents [12, 62, 100–103]. For that, differences in specificity or magnitude of local and systemic immune responses are expected. Also, local immune responses seem to be regulated jointly by the presence of microbial elicitors and endogenous signals from epithelial cells [104]. An interesting example is that of *Drosophila nasuta*, where the male transfers a substance within the ejaculate that would activate the phenoloxidase pathway leading to the formation of a large, opaque mass in the female’s uterus [105–106]. Here we find that the species that possess genital plug (i.e., those where the females must face a foreign body transferred by the male after mating) presented differences between the local and systemic immune response. In the *Urophonius* species the implants placed in the body cavity (systemic immune response elicited) were less encapsulated and more homogeneous than the genital implants (local immune response elicited). This would suggest a difference in the magnitude and specificity between the local and systemic immune response in these species. Conversely, *Z*. *fuscus*, the species without a genital plug, presented the same immunological parameters in the local and systemic cellular response. In general, the species did not differ in their systemic cellular immune responses, although implants with clearer NME encapsulations were observed in *Z*. *fuscus*. This does not necessarily mean a lower immune response at the systemic level in *Z*. *fuscus*, but perhaps interspecific differences in the effectiveness or time necessary for the melanization of implants. However, the interspecific differences in the local immune response in the female genital tract were very remarkable, which would suggest that the difference could be due to different agents or substances to which the genitalia is exposed (e.g., genital plugs, spermatophore structures, ejaculate accessory substances, specific microorganisms). In scorpions, the cuticle thickness and histological complexity of the female’s genital atrium have been related to mechanical damage caused by the capsular eversion of the spermatophore or by the introduction of genital plugs [32, 38, 76]. For instance, *Z*. *fuscus* presents simple spermatophores and a thin-walled genital atrium, whereas plug producing bothriurid species have more complex spermatophores and the genital atrium with folded epithelium and thick cuticular walls [38]. Besides, in several bothriurids, including *U*. *brachycentrus*, there have been seen regions in the atrium’s apical zone which contain epithelial cells with microvilli and pores connected to ducts [38, 76]. In addition, we observed a depletion in the THL after the genital implantation in the plug producing species, which was not observed in *Z*. *fuscus*. A change in the THL after an immunological challenge has been previously described in species of insects and crabs [107–113]. This change could be explained by the recirculation of free hemocytes in the hemolymph towards affected areas, where the phagocytic or encapsulating action of these cells are necessary, although future studies should focus on evaluating this possible mechanism.

### Female’s possible role in the formation of the *Urophonius* plug

We described inseminated females’ genital plugs of *U*. *achalensis* and *U*. *brachycentrus*. In all cases, the plugs blocked completely the lumen of the atrium, the genital aperture, and, in some cases one of the spermathecae’ ducts. Both the female and the male could be involved in the plug’s formation. The double conformation of the plug indicates a male’s contribution in that, the ‘initial plug’ transferred by the male is formed from the ‘hemi-plugs’ in each hemispermatophore when fused in the spermatophore (involving portions of the ejaculate and glandular products). The changes of the plug over time in morphology, size and coloration could indicate a female’s role. Our results have allowed us to rule out the hypothesis that these changes of the genital plugs were due to air contact or O_**2**_ influence. The *Urophonius*’ genital plugs present three different zones. Females of *U*. *achalensis* showed darker melanotic encapsulation in the ‘distal’ zone, and females of *U*. *brachycentrus* in the ‘middle’ zone. The ‘proximal’ zone of the genital plug does not present dark coloration, and coincidentally, this was the area in the genital implants with smaller melanotic encapsulations and clearer coloration. It was also found that the ‘distal’ and ‘proximal’ zones of the genital implants presented the larger non-melanotic encapsulations areas. The projections formed in the ‘distal’ zone of the genital implants extending below the genital operculum were similar in shape, size, and consistency to those found in the genital plugs. These results and the specificity of the local immune response of the plug producing species add evidence to the possible female’s role in the formation of portions of the plug.

### Types of identified hemocytes and quantified hemocyte load

We have described, for the first time, the types of hemocytes found in the female’s hemolymph in *Urophonius achalensis*, *U*. *brachycentrus* and, *Z*. *fuscus*. Two main types of cells were found: plasmatocytes (PLs) and granulocytes (GRs), in agreement with the findings of existing works on the subject [64, 95, 114–117]. Subtypes of hemocytes, Cystocytes (CYs), Spherulocytes (SPs) and Adipohemocytes (ADs), previously cited for scorpions [64, 95,114–117] were also identified. The existence of several types of hemocytes in the hemolymph would be an ancient character [118], which could have been retained in scorpions, one of the oldest arthropod groups ([119–122] but see [123]). There were not found prohemocytes, described as stem cells with embryonic nature [64, 95, 114, 117], probably due to their rapid conversion to other cell types [124]. Even though other subtypes of hemocytes, like oenocytoids or coagulocytes, were observed in scorpion species [64, 95, 114–115] there was not found any evidence of their presence in the species studied herein. *Zabius fuscus* had the highest values of THL compared those of plug producing species. The causes of this difference could be related to the evolutionary history of each species and the sexual and ecological context in which they have evolved [125–126]. Although the characteristics of the habitats are similar, the species present contrasting characteristics regarding patterns of surface activity at different times of the year [83–84]. In addition, these species could exhibit differences in microhabitats (Oviedo-Diego, Mattoni, Peretti personal observations), or other parameters such as diet or potential parasites [75].

### Correlations between immune parameters

Since multiple immunological parameters can be costly to maintain [25] trade-offs may exist between parameters within the same system. This would indicate an overlap in the resources used by different defense mechanisms, a cross-regulation between them or a common underlying mechanism [6, 29,125]. We found a negative correlation between the areas and the coloration of the encapsulations of the genital implants. This would suggest a trade-off between the encapsulation response *per se* (aggregation of layers of hemocytes) [127] and the melanization that occurs in these encapsulations (products generated by the phenoloxidase pathway) [8]. There was a close interrelationship between the humoral and cellular components [128], and some studies have reported antagonisms between the parameters of these systems, without investigating the underlying physiological mechanisms [89,129–130]. On the other hand, we found a positive correlation between body weight and THL. Variation in immunological parameters between individuals and species is expected [125,131] since the management of trade-offs between the costs of immune defense and other life history traits that overlap in the use of resources, can vary [26,132]. Heavier individuals may be more immunocompetent since they would have more circulating hemocytes ([133] but see [134]). However, it was also found that higher body weight individuals (*Z*. *fuscus* females) presented smaller areas of melanotic encapsulations on the genital implant and clearer encapsulations. A higher THL value would indicate a higher concentration of free hemocytes in hemolymph, and potentially a lower number of hemocytes in the genital area, resulting in less encapsulated and melanizated genital implants. It has also been reported that individuals with large numbers of hemocytes have a lower proportion of phagocytic hemocytes [135].

## Conclusions and perspectives of this study

These results help to elucidate some of the immune mechanisms triggered by the female against a foreign body in the reproductive tract. The study of the local immune response of encapsulation in the female genitalia is important to understand the plugging phenomenon in the two species of *Urophonius*. The description of the plug morphology and the cellular encapsulation of the genital implants have allowed us to observe complex structures and physiological processes. The encapsulation and melanization patterns on the genital implants may indicate greater and more specific response in females of species that have a genital plug. Future studies would evaluate the mechanisms underlying the observed immune responses. Sperm and other ejaculate accessory substances accompany the natural plug, and its formation will depend on multiple highly regulated factors and as a result of the interaction between the male and the female. In addition to this, the plugging occurs in the context of mating and multiple behavioral variables of mate evaluation, sex stimulation, or mating resistance cannot be ruled out, which can determine to some extent the characteristics of the plug. Therefore, next work will focus on the comparison of the immune response in females in the context of mating and taking into account the natural formation of genital plugs. Future and more detailed experiments will confirm the female role in the formation of the natural genital plugs of this species and the evolutionary interests underlying these post-copulatory mechanisms [47–48, 60–61]. We observed a depletion in the THL after the genital implantation in the plug producing species, which was not observed in *Z*. *fuscus*. Further studies would include analysis of changes in THL comparing virgin and inseminated females (with genital plug) to elucidate whether the observed results with genital implants actually reflect what happens when females are plugged. In addition, it is still necessary to corroborate whether the THL decrease could be due to recirculation of hemocytes with labeling methods or phagocytosis assay using Indian ink or fluorescent labelled bacteria. A first morphological description of the hemocytes has been made in these species, and in the future we plan to make more detailed descriptions, functional characterizations, inclusion of novel techniques such as genetic markers or antibodies, as well as other microscopy techniques such as scanning or electronic transmission, for the purpose of a precise classification, quantification and elucidation of the action mechanisms of these cells [115, 136–139]. Another interesting approach is a comparative and phylogenetic study of the plugs in the Bothriuridae Family what could provide information about the evolution of male and female strategies in terms of the plugging phenomenon, and whether this strategy, for example, is related in any way to other traits of the immune system or genital characters.

## Supporting information

S1 Table Mean values and standard deviations of different immunological parameters of *Urophonius brachycentrus*, *U*. *achalensis* and *Z*. *fuscus*. The melanotic (ME) and non-melanotic (NME) encapsulation response was measured in all three zones of implants placed in the body cavity (eliciting systemic immune response) and in the female genitalia (eliciting local immune response). The total hemocyte load (THL) before and after genital implantation, and the decrease in hemocyte concentration between both stages are presented. a, b, and c indicate the grouping and separation between stages and zones of the implant (p <0.05). Capital letters (A,B) indicate the grouping and separation between species (p <0.05) (i.e., interspecific comparison: reading vertically, not horizontally).
(DOCX)

S1 Fig Genital plugs and genital implants of the study species. (A) ‘Distal’ zone of genital plug below the female genital operculum of *Urophonius achalensis*. (B) Protruding distal portion of the genital implant positioned within the female genital atrium of *Urophonius achalensis*. (C) Implant inserted in the body cavity (in the membrane between the fifth and sixth segment of the mesosome) of *Z*. *fuscus*. (D) Genital plug extracted of an inseminated female of *Urophonius brachycentrus*. (E) Implant (nylon monofilament) before being placed on a female. (F) Scheme of a genital plug (*Urophonius*) and its positioning within the female genital atrium. (G) Scheme of a genital implant and its positioning within the female genital atrium. Abbreviations: ag, genital atrium; al, lumen of the genital atrium; bci, implant inserted in the body cavity (eliciting systemic immune reaction); bp, basal piece; Dz, distal zone; ga, genital aperture; gi, implant inserted in the genitalia (eliciting local immune reaction); gp, genital plug; ls, lumen of the seminal receptacle; ME, melanotic encapsulation; Mz, middle zone; NME, non-melanotic encapsulation; og, genital operculum; p, pectine; Pz, proximal zone; sr, seminal receptacle. Scale bars: 1 mm.
(TIF)

S2 Fig Implants eliciting local vs systemic immunological response in females of scorpion species. (A) Implant inserted in the body cavity of *Zabius fuscus* female. (B) Implant inserted in the body cavity of *Urophonius brachycentrus* female. (C) Implant inserted in the genitalia of *Z*. *fuscus* female. (D) Implant inserted in the genitalia of *U*. *achalensis* female. Abbreviations: bci, implant inserted in the body cavity (eliciting systemic immune reaction); Dz, distal zone; gi, implant inserted in the genitalia (eliciting local immune reaction); ME, melanotic encapsulation; Mz, middle zone; NME, non-melanotic encapsulation; Pz, proximal zone. Scale bars: 0.5 mm.
(TIF)
