# Supplementary material for: Specificity of the female’s local cellular immune response in genital plug producing scorpion species
Source: PLoS One. 2019 Feb 11;14(2):e0208682. doi: 10.1371/journal.pone.0208682 (PMC6370188; doi:10.1371/journal.pone.0208682)
Supplement: S1 Table — The melanotic (ME) and non-melanotic (NME) encapsulation response was measured in all three zones of implants placed in the body cavity (eliciting systemic immune response) and in the female genitalia (eliciting local immune response). The total hemocyte load (THL) before and after genital implantation, and the decrease in hemocyte concentration between both stages are presented. a, b, and c indicate the grouping and separation between stages and zones of the implant (p <0.05). Capital letters (A,B) indicate the grouping and separation between species (p <0.05) (i.e., interspecific comparison: reading vertically, not horizontally). (DOCX) [file pone.0208682.s001.docx]

| Immunological parameter | **Species** | **Encapsulation by zones of implant** | | | | | |
| --- | --- | --- | --- | --- | --- | --- | --- |
|  |  | Systemic immune response elicited | | | | | |
|  |  | *1.Distal* | | *2. Middle* | | *3. Proximal* | |
| ME area (mm^2^) | *U. brachycentrus* | 0.002 ± 0.003 | | 0.003 ± 0.002 | | 0.004 ± 0.006 | |
|  |  | a | A | a | A | a | A |
|  | *Z. fuscus* | 0.004 ± 0.003 | | 0.002 ± 0.001 | | 0.003 ± 0.002 | |
|  |  | a | A | b | A | b | A |
| NME area (mm^2^) | *U. brachycentrus* | 0.024 ± 0.010 | | 0.028 ± 0.012 | | 0.031 ± 0.015 | |
|  |  | a | A | a | A | a | A |
|  | *Z. fuscus* | 0.028 ± 0.015 | | 0.029 ± 0.020 | | 0.028 ± 0.010 | |
|  |  | a | A | a | A | a | A |
| ME coloration | *U. brachycentrus* | 44.836 ± 3.719 | | 40.318 ± 6.719 | | 48.520 ± 4.695 | |
|  |  | a | A | b | A | a | A |
|  | *Z. fuscus* | 43.052 ± 6.025 | | 35.125 ± 4.014 | | 38.200 ± 8.338 | |
|  |  | a | A | a | A | a | A |
| NME coloration | *U. brachycentrus* | 97.857 ± 16.243 | | 98.761 ± 8.558 | | 93.740 ± 8.764 | |
|  |  | a | B | a | B | a | B |
|  | *Z. fuscus* | 121.487 ± 19.006 | | 129.423 ± 23.581 | | 123.145 ± 14.422 | |
|  |  | a | A | a | A | a | A |
|  | | Local immune response elicited | | | | | |
|  |  | *1.Distal* | | *2. Middle* | | *3. Proximal* | |
| ME area (mm^2^) | *U. achalensis* | 0.073 ± 0.017 | | 0.008± 0.005 | | 0.022 ± 0.004 | |
|  |  | a | A | b | A | b | A |
|  | *U. brachycentrus* | 0.024 ± 0.012 | | 0.004 ± 0.002 | | 0.003 ± 0.002 | |
|  |  | a | B | ab | A | b | A |
|  | *Z. fuscus* | 0.004 ± 0.003 | | 0.001 ± 0.001 | | 0.002 ± 0.002 | |
|  |  | a | B | b | B | b | B |
| NME area (mm^2^) | *U. achalensis* | 0.06 ± 0.027 | | 0.034 ± 0.011 | | 0.071 ± 0.028 | |
|  |  | a | AB | b | A | a | A |
|  | *U. brachycentrus* | 0.04 ± 0.015 | | 0.026 ± 0.012 | | 0.063 ± 0.017 | |
|  |  | a | A | b | A | a | A |
|  | *Z. fuscus* | 0.032 ± 0.007 | | 0.03 ± 0.011 | | 0.026 ± 0.011 | |
|  |  | a | B | a | A | a | A |
| ME coloration | *U. achalensis* | 30.647 ± 3.949 | | 35.536 ± 0.92 | | 33.917 ± 2.545 |  |
|  |  | b | A | a | A | a | A |
|  | *U. brachycentrus* | 40.18 ± 3.958 | | 36.53 ± 4.684 | | 43.336 ± 2.51 |  |
|  |  | b | B | c | A | a | B |
|  | *Z. fuscus* | 42.843 ± 2.526 | | · | | · |  |
|  |  |  |  |  |  |  |  |
| NME coloration | *U. achalensis* | 87.65 ± 13.282 | | 93.295 ± 24.385 | | 86.9135 ± 14.827 |  |
|  |  | a | A | a | A | a | A |
|  | *U. brachycentrus* | 89.516 ± 22.496 | | 90.841 ± 17.245 | | 93.324 ± 19.121 |  |
|  |  | a | A | a | A | a | A |
|  | *Z. fuscus* | 100.03 ± 11.951 | | 126.924 ± 16.341 | | 122.436 ± 16.049 |  |
|  |  | b | A | a | B | ab | A |
|  | | **Total hemocyte load (THL)** | | | | |  |
|  |  | *Pre-implantation* | | *Post-implantation* | | *Diminution (%)* | |
| Number hemocytes/ml hemolymph | *U. achalensis* | 2.90E+06 ± 8.90E+05 |  | 1.94E+06 ± 7.20E+05 |  | 33.121 |  |
|  |  | a | B | b | C |  | B |
|  | *U. brachycentrus* | 5.37E+06 ± 1.26E+06 |  | 2.92E+06 ±1.24E+06 |  | 45.517 |  |
|  |  | a | B | b | B |  | A |
|  | *Z. fuscus* | 1.19E+07 ± 2.61E+06 |  | 1.15E+07 ± 2.88E+06 |  | -11.708 |  |
|  |  | a | A | a | A |  | · |
