# Supplementary figures and images for: Specificity of the female’s local cellular immune response in genital plug producing scorpion species
Source: PLoS One. 2019 Feb 11;14(2):e0208682. doi: 10.1371/journal.pone.0208682 (PMC6370188; doi:10.1371/journal.pone.0208682)

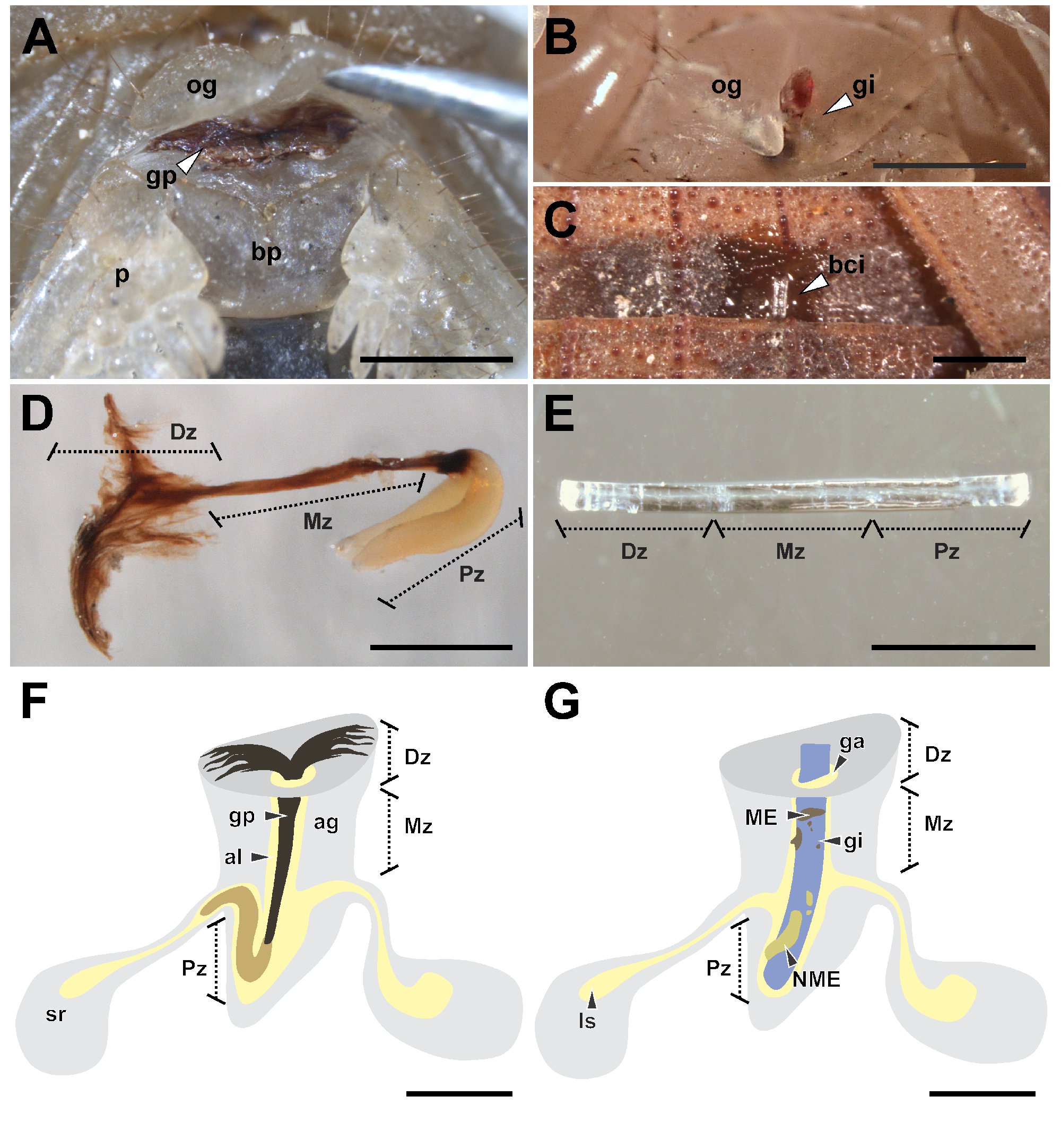

Supplement: S1 Fig — (A) ‘Distal’ zone of genital plug below the female genital operculum of Urophonius achalensis. (B) Protruding distal portion of the genital implant positioned within the female genital atrium of Urophonius achalensis. (C) Implant inserted in the body cavity (in the membrane between the fifth and sixth segment of the mesosome) of Z. fuscus. (D) Genital plug extracted of an inseminated female of Urophonius brachycentrus. (E) Implant (nylon monofilament) before being placed on a female. (F) Scheme of a genital plug (Urophonius) and its positioning within the female genital atrium. (G) Scheme of a genital implant and its positioning within the female genital atrium. Abbreviations: ag, genital atrium; al, lumen of the genital atrium; bci, implant inserted in the body cavity (eliciting systemic immune reaction); bp, basal piece; Dz, distal zone; ga, genital aperture; gi, implant inserted in the genitalia (eliciting local immune reaction); gp, genital plug; ls, lumen of the seminal receptacle; ME, melanotic encapsulation; Mz, middle zone; NME, non-melanotic encapsulation; og, genital operculum; p, pectine; Pz, proximal zone; sr, seminal receptacle. Scale bars: 1 mm. (TIF) [file pone.0208682.s002.tif]

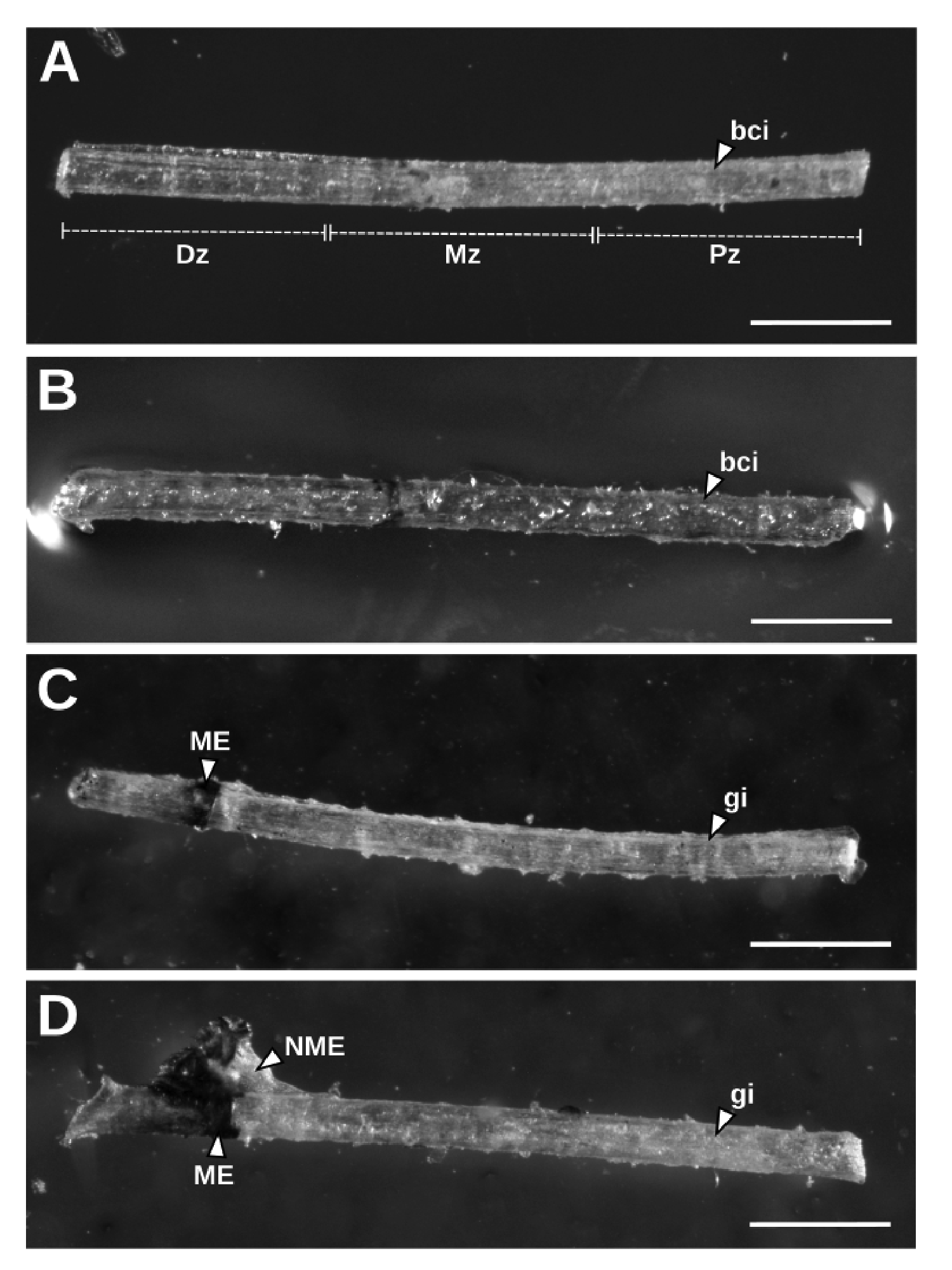

Supplement: S2 Fig — (A) Implant inserted in the body cavity of Zabius fuscus female. (B) Implant inserted in the body cavity of Urophonius brachycentrus female. (C) Implant inserted in the genitalia of Z. fuscus female. (D) Implant inserted in the genitalia of U. achalensis female. Abbreviations: bci, implant inserted in the body cavity (eliciting systemic immune reaction); Dz, distal zone; gi, implant inserted in the genitalia (eliciting local immune reaction); ME, melanotic encapsulation; Mz, middle zone; NME, non-melanotic encapsulation; Pz, proximal zone. Scale bars: 0.5 mm. (TIF) [file pone.0208682.s003.tif]
